# Prevalence and Outcome of Secondary Hemophagocytic Lymphohistiocytosis Among SIRS Patients: Results from a Prospective Cohort Study

**DOI:** 10.3390/jcm8040541

**Published:** 2019-04-19

**Authors:** Guido A. Gualdoni, Georg A. Hofmann, Philipp Wohlfarth, Heide-Maria Winkler, Stefan Winkler, Helmuth Haslacher, Renate Thalhammer, Athanasios Makristathis, Franz Ratzinger, Heinz Burgmann

**Affiliations:** 1 Division of Infectious Diseases and Tropical Medicine, Department of Medicine I, Medical University of Vienna, 1090 Vienna, Austria; guido.gualdoni@meduniwien.ac.at (G.A.G.); n1203609@students.meduniwien.ac.at (G.A.H.); heide-maria.winkler@meduniwien.ac.at (H.-M.W.); stefan.winkler@meduniwien.ac.at (S.W.); heinz.burgmann@meduniwien.ac.at (H.B.); 2 Division of Nephrology and Dialysis, Department of Medicine III, Medical University of Vienna, 1090 Vienna, Austria; 3 Division of Bone Marrow Transplantation, Department of Medicine I, Medical University of Vienna, 1090 Vienna, Austria; Philipp.wohlfarth@meduniwien.ac.at; 4 Department of Laboratory Medicine, Medical University of Vienna, 1090 Vienna, Austria; Helmuth.haslacher@meduniwien.ac.at (H.H.); renate.thalhammer@meduniwien.ac.at (R.T.); Athanasios.Makristathis@meduniwien.ac.at (A.M.)

**Keywords:** hemophagocytic lymphohistiocytosis, HLH, hemophagocytosis, SIRS, survival, outcome, prevalence

## Abstract

Secondary hemophagocytic lymphohistiocytosis (sHLH) is a life-threatening condition clinically presenting as SIRS (Systemic Inflammatory Response Syndrome). However, there is no comprehensive data concerning diagnostic algorithms, prevalence, outcome and biomarker performance in SIRS patients. We conducted a prospective observational cohort study on 451 consecutive patients fulfilling ≥2 SIRS criteria. The Hscore and the HLH-2004 criteria were used to determine the presence of sHLH, and the correlation of the screening-biomarkers ferritin, sCD25, and sCD163 with both scores was assessed. Out of 451 standard-care SIRS patients, five patients had high Hscores (≥169), suggesting incipient or HLH-like disease, and these patients were in urgent need for intensified therapy. However, none of these patients fulfilled five HLH-2004 criteria required for formal diagnosis. From the studied biomarkers, ferritin correlated strongest to both the HLH-2004 criteria and the Hscore (rs = 0.72, 0.41, respectively), and was the best predictor of 30-day survival (HR:1.012 per 100 μg/L, 95% CI: 1.004–1.021), when adjusted for patient’s age, sex, bacteremia and malignant underlying-disease. Also, the HLH-2004 (HR per point increase: 1.435, 95% CI: 1.1012–2.086) and the Hscore (HR per point increase:1.011, 95% CI: 1.002–1.020) were independent predictors of 30-day-survival. The Hscore detected patients in hyperinflammatory states requiring urgent therapy escalation. Degrees of hyperinflammation, as assessed by ferritin and both HLH scores, are associated with worse outcomes.

## 1. Background

Hemophagocytic lymphohistiocytosis (HLH) is a rare but potentially lethal disorder characterized by erratic immune activation [1]. Currently, hereditary or primary forms of the condition are distinguished from secondary or reactive forms [2,3,4]. Hereditary HLHs affect children and are caused by mutations in genes of the cytotoxic granule complex, resulting in defective pathogen elimination and excessive immune activation due to persistent stimuli [5]. In contrast, reactive or secondary HLH (sHLH) more frequently affects adults in response to an infectious stimulus on the ground of a predisposing condition usually involving immune-dysfunction, e.g., malignant hematologic or rheumatic disorders [6,7].

Diagnosis of both HLH forms is based on the HLH-2004 criteria (summarized Supplementary Table S1) [8]. However, these criteria were primarily designed for the detection of hereditary HLH, and there have been concerns both regarding the applicability since some diagnostic features are not easily accessible in clinical practice (i.e., sCD25, NK cell activity measurement) and they appear to be of limited clinical utility particularly in adult autoimmune diseases [9]. Therefore, Fardet and colleagues developed a scoring system termed “Hscore” for a more straightforward assessment of HLH features focusing on the acquired, sHLH subgroup [10]. In contrast to the HLH-2004 criteria, the Hscore does not per se justify a diagnosis, but calculates the probability of sHLH presence.

Several biomarkers have been proposed to facilitate diagnosis of this complex syndrome, the most established ones being sCD25 [11] and ferritin [12,13] (which are both components of the mentioned scores), and the soluble hemoglobin-haptoglobin scavenger receptor CD163, which was proposed to add specificity due to its origin from activated macrophages [14,15].

Despite these rigorous efforts to guide clinicians in diagnosing sHLH, the discrimination of this entity from other conditions, particularly sepsis, remains challenging due to the large degree of overlap in clinical presentation [16,17]. Further complicating clinical diagnosis, our current knowledge considering prevalence, presentation, and disease course is based on either small case series, case reports or specific patient subgroups [7,12,18,19,20].

In this study, we aimed to assess the prevalence and disease course of sHLH among SIRS (Systemic Inflammatory Response Syndrome) patients on standard care wards at a large tertiary care hospital. We illustrate the relationship between the HLH-2004 criteria and the Hscore in this patient cohort and assess the diagnostic capacity of the most established serological biomarkers ferritin, sCD25 and sCD163 for diagnosis in this setting. Since some authors have proposed to regard sHLH as the maximal form in a spectrum of hyperinflammation [21], we analyzed whether the HLH-2004 criteria and the Hscore had an impact on survival in our cohort, even in a non-HLH suggestive scale.

## 2. Materials and Methods

### 2.1. Study Design

The present study was conducted within a prospective observational cohort study on SIRS patients. The primary objectives of this study were to establish biomarkers, potential complications and machine learning algorithms for diagnosis of SIRS [22,23,24]. The patient recruitment phase of this study was conducted between July 2011 and September 2012 on 27 standard care wards (13 surgical and 14 medical) at the Vienna General Hospital, Austria, a large tertiary care hospital. The study plan was approved by the ethics committee of the Medical University of Vienna (EC-No. 518/2011) and the study was conducted in accordance with the Declaration of Helsinki 1964 (including current revisions). All participants gave oral and written informed consent.

All patients with clinical suspicion of bacterial infection and for whom blood cultures were requested at one of the participating wards were screened for the following inclusion criteria: (1) at least two SIRS criteria as in [25], (2) at least 18 years, and (3) ability to give consent. Iatrogenically induced neutropenia was not considered an applicable SIRS criterion. Exclusion criteria were (1) less than 72 h after surgery (postoperative fever), (2) infection with HIV, or (3) inability to assign the patient to a predefined outcome group (infection vs. non-infection associated SIRS syndrome). For details, see [22].

Patients were followed-up during the hospital stay in which study inclusion took place. Long-term survival data (1095 days) was obtained for all participants by an inquiry to the Austrian Federal Statistical Office.

### 2.2. Data Collection and Laboratory Analyses

Clinical data were prospectively collected during patients’ recruitment and were completed after hospital discharge as described above. For details concerning microbiological analysis and analysis of routine parameters see Supplementary Information.

Both sCD25 and sCD163 were analyzed in all patients from plasma collected at the first day after blood culture taking with DuoSet® ELISA from R&D Systems (Minneapolis, MN, USA) according to the manufacturer’s protocol. Used biospecimens were processed and stored until analysis at the Medical University of Vienna Biobank Facility. Standard sample processing procedures were published previously [26]. For sCD25, the NIBSC 97/600 standard (NIBSC, Blanche Lane, UK) was used to calculate International Units. Bone marrow biopsy and imaging were performed as clinically indicated, and if available, results which were obtained in the two weeks prior to or after the onset of SIRS were utilized to address hemophagocytosis and hepatosplenomegaly, respectively. Examination of bone marrow specimens was available in 34 patients. Imaging of liver and spleen was available in 237 patients.

### 2.3. Statistical Analysis

For statistical analysis R (R Foundation for Statistical Computing, Version 3.3.0, Vienna, Austria) was used. Group differences were compared by using the Kruskal–Wallis test or Fisher’s exact test. Furthermore, the Spearman’s rank correlation coefficient (rs) was used. The maximally selected rank statistics (maxstat) method was applied, to binarize the HLH-2004 criteria and the Hscore [27]. For a graphical representation of patients’ survival, Kaplan-Maier plots were used and survival curves were compared by using the logrank test. Moreover, Cox proportional hazards models were applied for the evaluating the impact of the HLH-2004 criteria and the Hscore on the patient’s survival probability, when adjusted for patient’s age, sex, bacteremia and presence of malignant disease. Statistical significance is specified as *p*-values less than 0.05 (two-tailed). The Bonferroni-Holm correction was applied to correct for an alpha accumulation error related to multiple testing.

## 3. Results

### 3.1. Demographic Data

In total, 451 prospectively recruited SIRS patients were available for this evaluation. Among these, 126 patients (27.9%) suffered from bacteremia, 191 patients (42.4%) suffered from infection without evidence of bacteremia and 134 patients (29.7%) presented with a SIRS syndrome but without evidence of an on-going infection. Baseline characteristics are summarized in Table 1. Supplementary Table S2 presents the distribution of infections according to ECDC classification criteria established for point prevalence studies.

### 3.2. Features of Hemophagocytic Syndrome

**Figure 1:**
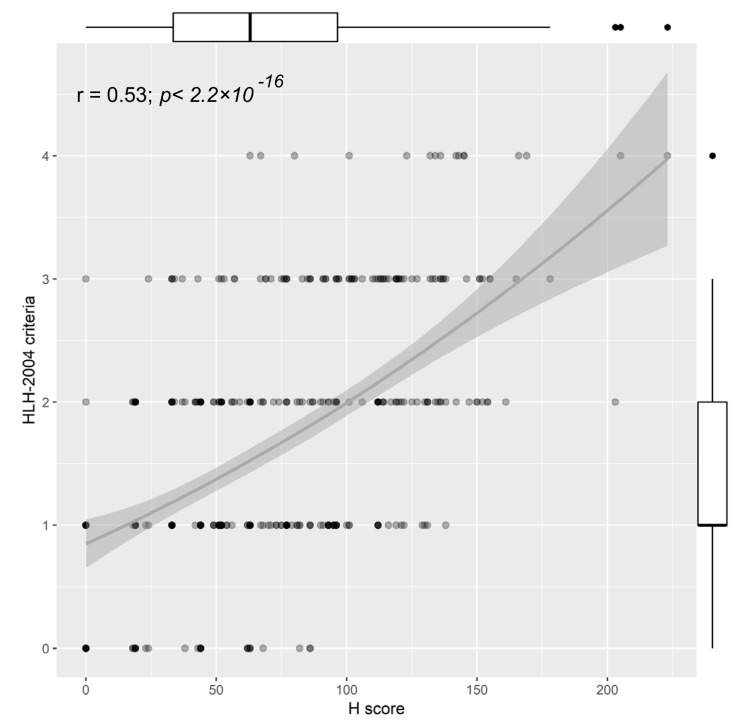
Correlation between the HLH-2004 criteria and the Hscore; Boxplots of the corresponding score are displayed on the opposite side of the plot, r = Spearman rank correlation coefficient.

The study population presented with a median of 1 HLH-2004 criterion (Q1–Q3: 1–2), and a median Hscore of 63.0 (Q1–Q3: 33.5–96.5). Both hemophagocytosis scores correlated only in a moderate range (rs = 0.53, Figure 1 and Supplementary Figure S1). Interestingly, we found the weakest correlation between the scores in younger women (<63 years, rs = 0.41, Supplementary Figure S1).

While none of the patients was found with more than 5 HLH-2004 criteria, five patients (prevalence: 1.1%) presented with an Hscore of ≥169 points (see Table 2).

The highest Hscore (223 points, >96% HLH probability) was found in patient ID 38, a 59-year-old female suffering from acute refractory lymphoblastic leukemia for 12 months. Despite receiving induction therapy and re-induction therapies, no remission was achievable, and therefore, a bridging therapy to stem cell transplantation was given. During that bridging therapy, the patient developed severe pancytopenia with SIRS symptoms and died 15 days after the first onset of SIRS symptoms with having Pseudomonas aeruginosa bacteremia.

Patient ID 288 was a 21-year-old female (Hscore: 205, >88% HLH probability) who presented to the emergency department with fever, a rash and elevated signs of inflammation. Despite empiric antibiotic treatment with ampicillin/sulbactam, the patient’s condition worsened with on-going fevers, high signs of inflammation, elevated transaminases, and hepatosplenomegaly. No infectious agent could be identified in repeatedly taken blood culture samples. Eventually, the patient received high-dose corticosteroids for a suspected underlying rheumatologic disorder. Thereon, her condition resolved and she was rapidly discharged. The condition was later identified to be adult-onset Still’s disease, and the patient experienced several episodes of this kind until she died at the age of 27 years due to a therapy-refractory sHLH (6/8 HLH-2004 criteria, NK cell activity, and sCD25 not assessed).

ID 573 is a 32-year-old female (Hscore: 203, >88% HLH probability), who had received double lung transplantation related to chronic thromboembolic pulmonary hypertension (CTEPH) two years previously. The SIRS episode was accompanied by massive upper abdominal pain, fatigue and weakness with hepatomegaly and hepatopathy of unclear genesis (significant cholestasis, increased γGT, and bilirubin), pancreatitis, and acute renal failure. In liver biopsy, a non-necrotizing granulomatous inflammation consistent with vasculitis was found; however, the etiology remained unclear. Moreover, in the blood culture, Enterococcus faecalis and Staphylococcus epidermidis were detected. The patient survived this episode after receiving piperacillin as well as high-dose steroids but died later on (>1 year) due to her underlying disease.

Patient ID 726 was a 45-year-old female (Hscore: 178, >70% HLH probability), who was admitted to the hospital due to suspected systemic lupus erythematosus with massive oedematous swelling of the face, fever, skin rashes, arthralgia, fatigue and neurologic symptoms (psychosis). In addition to an increase in creatinine, creatinine kinase, and liver transaminases, antibodies against double-stranded DNA with an increase in IgG, as well as reduced complement factors, were also found. In renal biopsy, lupus-nephritis was observed. The patient received repeated immune-apheresis and therapy with high-dose steroids, chloroquine and azathioprine. Upon therapy, both her clinical and laboratory alterations resolved.

ID 87 (Hscore: 169, ≈50% HLH probability) was a 65-year-old man suffering from acute myelomonocytic leukemia, which he had developed additionally to a pre-existing chronic lymphatic leukemia. After administration of induction chemotherapy and two consolidation therapies, a relapse was observed. At the beginning of the SIRS symptoms, the patient showed increased inflammatory parameters and recurrent fever attacks up to 39.4 °C. The patient died two weeks after onset.

As this study was designed as an observational study, none of the treating physicians was aware of the scores reported here and none of the patients in this study received a specific therapy targeting sHLH as a differentiated entity.

### 3.3. Survival Data

Within the study population, the in-hospital mortality was 11.5% (*n* = 52). The association between hemophagocytosis scores, biomarkers and mortality were both evaluated after 30 days (short-term survival to exclude a potential impact of the underlying disease) and after a longer timeframe (between 31 and 1095 days).

**Figure 2:**
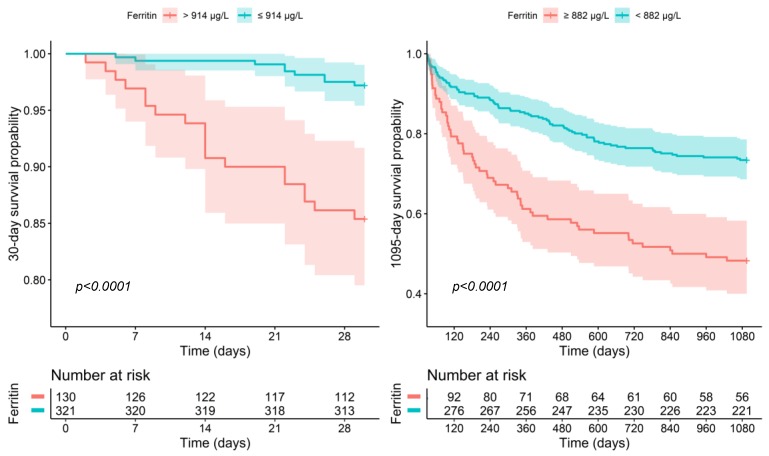
30-day and 1095-day survival with respect to ferritin.

For 30-day survival, both the HLH-2004 criteria (HR per point increase: 1.435, 95% CI: 1.012–2.086, *p* = 0.043) and the Hscore (HR per point increase: 1.011, 95% CI: 1.002–1.020, *p* = 0.018) were independently associated with increased mortality, when adjusted for patient’s age, sex and presence of bacteremia or a malignant disease. The strongest association with worse outcomes was found for ferritin (HR per 100 μg/L: 1.012, 95% CI: 1.004–1.021, *p* = 0.006) and sCD25 (HR per 10 IU/mL: 1.026, 95% CI: 1.007–1.045, *p* = 0.006), when adjusted for patient’s age, sex and presence of bacteremia or a malignant disease. In contrast, sCD163 did not show an association (adjusted HR: 1.008, 95% CI: 1.000–1.017, *p* = 0.050). 914.3 μg/L ferritin or 104.5 IU/mL sCD25 were identified as optimal cut-off point resulting in an HR of 0.178 (95% CI: 0.081–0.394; *p* < 0.001, see: Figure 2) or 0.373 (95% CI: 0.178–0.783, *p* = 0.009) for the low risk group.

For long-term survival, ferritin (HR: 1.011 per 100 μg/L, 95% CI: 1.006–1.017, *p* < 0.0001) and sCD163 (HR: 1.009 per 10 pg/mL, 95% CI: 1.005–1.013, *p* < 0.0001) were the best predictors for a decreased outcome, when adjusted for age, sex and presence of bacteremia or a malignant disease. The Hscore (HR per point increase: 1.005, 95% CI: 1.001–1.009, *p* = 0.027) or the HLH-2004 criteria (HR per point increase: 1.222, 95% CI: 1.031–1.448, *p* = 0.021), both showed a moderate association with worse outcome, while sCD25 showed no significant association (HR: 1.007 95% CI: 0.994–1.020 per 10 IU increase, *p* = 0.2831). Patients with less than 881.5 μg/L ferritin or 1057.3 pg/mL sCD163 had a significantly better long-term survival (HR: 0.416, 95% CI: 0.297–0.582, *p* < 0.001, see Figure 2; HR: 0.462, 95% CI: 0.326–0.656, *p* < 0.001, respectively).

### 3.4. Prediction of Hemophagocytosis Scores and Utility of Biomarkers

**Figure 3:**
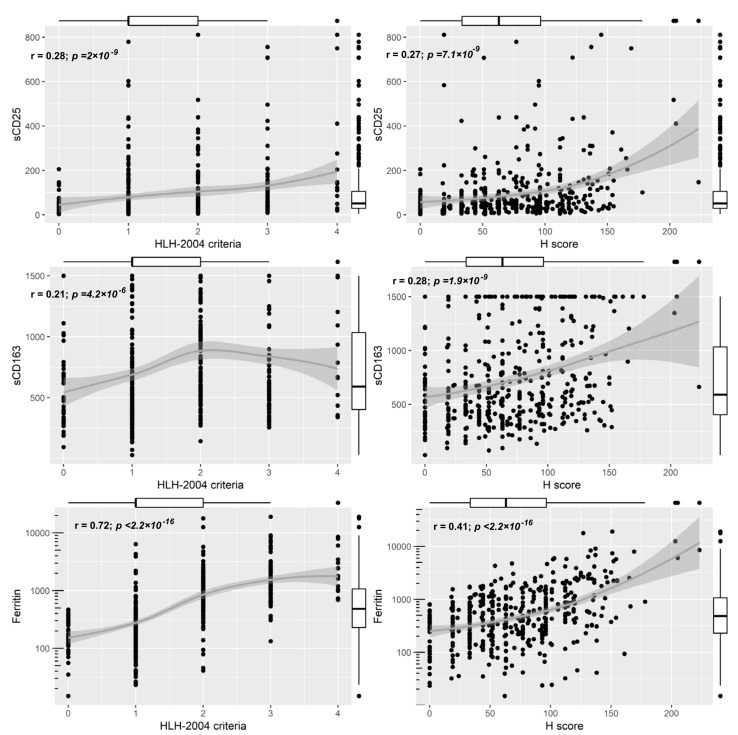
Correlations between sCD25, sCD163 and Ferritin the studied HLH-scores; Boxplots of the corresponding score are displayed on the opposite side of the plot, r = Spearman rank correlation coefficient.

To assess possible screening parameters for sHLH in SIRS patients, we analyzed the correlation of sCD25, sCD163 and ferritin to the studied HLH-scores. sCD25 and sCD163 showed only a poor correlation with both hemophagocytes scores (rs ranged between 0.21–0.28, Figure 3). Ferritin correlated better to both the HLH-2004 criteria and the Hscore (rs = 0.72, rs = 0.41, respectively; Figure 3). The correlation between ferritin and the HLH-2004 criteria was comparable between age and sex groups, whereas the correlation between ferritin and the Hscore was strongest in older (≥63 years) females and younger (≤63 years) males (Supplementary Figure S2).

## 4. Discussion

In the absence of precise data concerning sHLH among SIRS patients, the present study aimed to assess the prevalence, disease course and outcome of this entity in this setting. Furthermore, we investigated the utility of the best-established biomarkers in these patients. To our knowledge, this is the first large prospective study addressing this matter.

Interestingly, we found a weak correlation between the HLH-2004 criteria and the Hscore. The HLH-2004 criteria were initially designed for the diagnosis of primary HLH in a pediatric patient population, and there has been a debate about the utility of this scoring system for an sHLH population [12]. In contrast to the HLH-2004 criteria, the Hscore detected patients with an urgent need for the establishment of aggressive therapy, although the treating physicians were not aware of the score (which was not available at the time of recruitment). Consensus needs to be found concerning the diagnostic criteria for sHLH, since the discrepancies between diagnostic algorithms might impair the rapid initiation of specific therapy.

Overall, we found five patients in our SIRS cohort (prevalence: 1.1%) with a relevant elevation in the Hscore, suggesting incipient or HLH-like disease. Of these five patients, two had a malignant hematologic disease and three had an autoimmune disease, which is in line with previous reports describing these diseases to predispose for sHLH development [12,19,28,29]. In a retrospective population-based study, Machaczka and colleagues found an incidence of 0.9% of HLH among individuals with malignant diseases in Sweden [29]. In our study, a total of 177 SIRS patients with malignant diseases were enrolled, resulting in a prevalence of 1.1% (2/177) of HLH-like disease among patients with malignant diseases in our cohort.

In works assessing the prevalence of “hyperinflammatory sepsis” (also termed sepsis with macrophage-activation like syndrome (MALS) or features of macrophage-activation syndrome) in critical care settings, higher prevalence (3.7–5.6%) was found than in our study [30,31]. These discrepancies are mostly driven by different classification criteria, since the presence of hepatobiliary dysfunction, disseminated intravasal coagulation or an elevated Hscore with a lower cut-off were considered sufficient for grading as MALS. We chose to apply the more stringent classification criteria, as we were interested in assessing the prevalence of sHLH in this cohort. Furthermore, the mentioned studies recruited patients mainly on ICUs, whereas the recruitment in our study was performed exclusively on standard care wards. Of note, we found a significant increase in short term mortality in patients with higher Hscores and HLH-2004 criteria, which indicates that hyperinflammation, as assessed by these scores, is associated with worse outcomes. Nonetheless, these findings outline the necessity to clearly define these entities in order to pave the ground for future intervention studies and thus to distinguish patients that might benefit from specific therapies (e.g., IL-1 blockade [6,30]). In our cohort, ferritin performed better than both the Hscore and the HLH-2004 criteria for the prediction of short- and long- term mortality. This is particularly intriguing, considering that ferritin is a component of both scoring systems. Similarly, Kyriazopoulou et al. reported 66% increased short-term mortality in patients with ferritin above 4420 ng/mL [31]. These and our findings emphasize considerations of other authors to place hyperferritinemia at the center of the classification of hyperinflammatory entities [32].

In-hospital mortality among the identified patients with HLH-like disease was higher than in the general cohort (40% vs. 11.5%), but was highly dependent on the underlying cause (0/2 survived with underlying hematological disorders, whereas 3/3 survived with rheumatological disorders). These findings underline considerations of other authors that sHLH is a heterogenic condition probably requiring differentiated therapy [33].

Infectious agents have been shown to be of outstanding importance in triggering reactive sHLH [34]. In three out of the five HLH-like cases of our cohort, there was an infectious trigger found, resulting in immune-dysregulation. Again, this underlines previous reports describing the high abundance of infectious agents triggering sHLH exacerbations [6,34,35].

Furthermore, we found that ferritin showed a rank correlation coefficient of 0.72 with the HLH-2004 criteria. All five patients with HLH-like disease had hyperferritinemia of >500 μg/L (cut-off HLH-2004 criteria). Similar to other authors, we found most values of ferritin to be far above the HLH-2004 cut-off in patients with HLH-like disease [36], and these authors have suggested that higher cut-offs might improve the utility of this marker in sHLH diagnosis. Nevertheless, the value of hyperferritinemia in the diagnosis of HLH has been a matter of debate lately. Early studies have promoted the utilization of serum ferritin for diagnosis and prognostic evaluation in these patients [12,37,38]. However, more recent data has outlined the low specificity of ferritin values even as high as >50.000 μg/L in adults [13,39]. Notably, neither sCD163 nor sCD25 showed a good correlation with any of the two HLH-scores studied. This is in contrast to recent studies, showing good specificity and sensitivity of high sCD25 [11,40] and sCD163 values [14]. The discrepancies in our findings concerning the studied biomarkers might also be a result of the early analysis at day 1 after SIRS onset, whereas the majority of earlier studies have studied biomarker expression after a clinical suspicion for the presence of sHLH had triggered a broader diagnostic work-up. Given the high dynamics of the syndrome, repeated testing of these biomarkers and evaluations in the context of the clinical manifestation might be advisable. However, our data question the utility of sCD25, sCD163 and ferritin as single parameters for early sHLH-screening in standard care SIRS-patients.

A limitation of our study is that bone marrow samples and assessment of hepatosplenomegaly were performed only when clinically indicated. Bone marrow biopsy samples were available in 34 of cases and imaging of liver and spleen in 237 of cases. Consequently, this study might underestimate the prevalence of the condition in this patient cohort, since a criterion of the HLH-2004 criteria and Hscore might have been missed. However, studies have shown a poor correlation between hemophagocytosis in the bone marrow and a clinically relevant HLH, since features of incipient hemophagocytosis might also be found in non-affected individuals [19,41,42,43]. Hence, the role of bone marrow biopsy in the diagnosis of sHLH is increasingly a matter of debate [7]. Furthermore, our approach is in line with current clinical practice, and an in-depth screening including not-indicated investigations would not comply with ethical considerations.

In our setting, the Hscore was more efficient than the HLH-2004 criteria in the detection of patients with an urgent need for intensified therapy. Degrees of hyperinflammation, as assessed by both scores, were associated with worse outcomes. No single biomarker was suitable for screening purposes. Further studies are needed in order to further establish the best diagnosis, screening and treatment concepts for these patients.

## Figures and Tables

**Table 1:** Clinical data of the study population.

Feature	SIRS = 2 (*n* = 181)	SIRS = 3 (*n* = 201)	SIRS = 4 (*n* = 69)	*p*–Value
Age	57.9 (44.4–69.0)	58.6 (42.5–69.0)	56.1 (40.7–69.9)	0.946
Male:Female	42%:58%	45%:55%	44%:56%	0.806
Immuno-suppression	28 (16%)	27 (13%)	10 (15%)	0.857
Neoplasm	63 (35%)	84 (42%)	30 (44%)	0.275
sCD25 (IU/mL)	47.5 (26.7–93.3)	54.7 (28.2–108.9)	62.2 (36.4–146.1)	0.091
sCD163 (pg/mL)	610.9 (381.7–1022.5)	572.8 (403.3–1046.5)	631.5 (475.5–1036.3)	0.404
HLH-2004 criteria	1.0 (1.0–2.0)	2.0 (1.0–3.0)	2.0 (1.0–3.0)	<0.001 *
Hscore	63.0 (33.0–95.0)	67.0 (33.0–101.0)	75.0 (44.0–112.0)	0.212

* significant after applying the Bonferroni-Holm correction, *p*-values are calculated using the Kruskal-Wallis test or Fisher’s exact test; Immunosuppression = clinically significant immunosuppression induced by therapeutic intervention. SIRS–Systemic Inflammatory Response Syndrome.

**Table 2:** Data of patients with high Hscores.

ID		38	288	573	726	87
Hscore		223	205	203	178	169
HLH Probability*		>96%	>88%	>88%	>70%	~50%
HLH-2004 Criteria		4	4	2	3	3
Temp (°C)		38.5	40.2	40	38.5	38.7
Cytopenia	Hb (g/dL)	6.8	10.4	9.1	9.2	9.8
	Platelets (G/L)	34	105	166	110	8
	WBC (G/L)	0.08	13.79	6.65	2.47	15.75
No of Cytopenia		3	1	1	1	1
Spleno/Hepatomegaly		HM	SM	HM	n.a.	SM
Immunosuppression		no	no	yes	yes	no
Triglycerides (mg/dL)		387	360	252	285	184
Fibrinogen (mg/dL)		705	359	687	227	512
Ferritin (μg/L)		8577	6053	12582	903	7950
ASAT/GOT (IU/L)		80	190	81	640	127
Hemophagocytosis		no	n.a.	n.a.	n.a.	n.a.
sCD25 (IU/mL)		147.4	410.8	518.0	101.1	749.9
sCD163 (pg/mL)		661.92	>1500	1348.2	>1500	>1500

* according to the Hscore; n.a. = not assessed, HM = Hepatomegaly; SM = Splenomegaly.

## Supplementary Materials

The following are available online at https://www.mdpi.com/2077-0383/8/4/541/s1. Figure S1: Long-term survival of SIRS patients in respect to studied HLH-scores. Figure S2: Age-and sex-dependent biomarker correlation to the studied scores. Table S1: Overview of the HLH-2004 criteria and Hscore criteria. Table S2: Distribution of infections according to ECDC classification criteria modified ECDC class. Supplementary Methods.
