# Supplementary material for: Prevalence and Outcome of Secondary Hemophagocytic Lymphohistiocytosis Among SIRS Patients: Results from a Prospective Cohort Study
Source: J Clin Med. 2019 Apr 19;8(4):541. doi: 10.3390/jcm8040541 (PMC6518152; doi:10.3390/jcm8040541)
Supplement: Supplementary file 1 [file jcm-08-00541-s001.pdf]

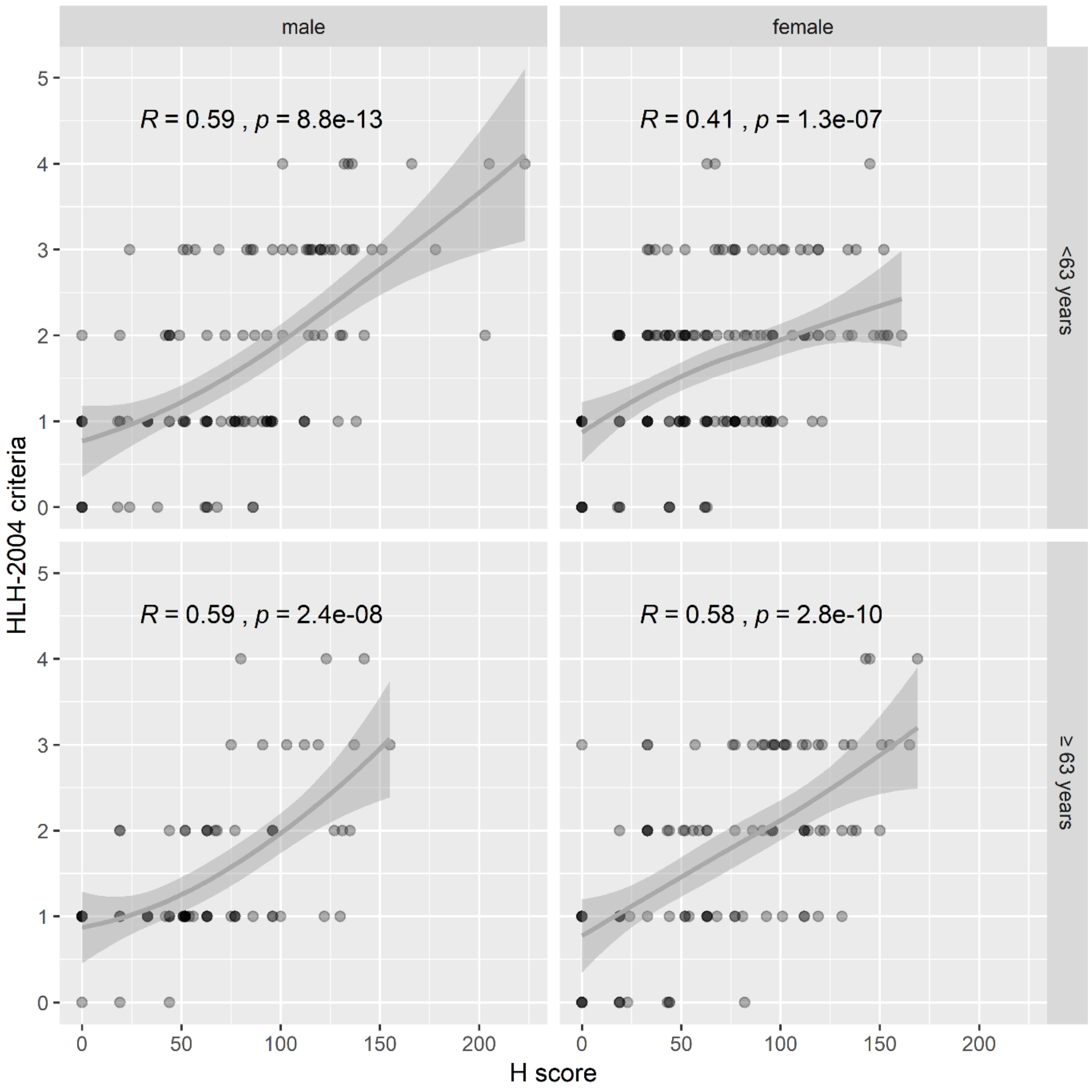

Figure S1. Age- and sex-dependent correlation between the Hscore and the HLH-2004 criteria.

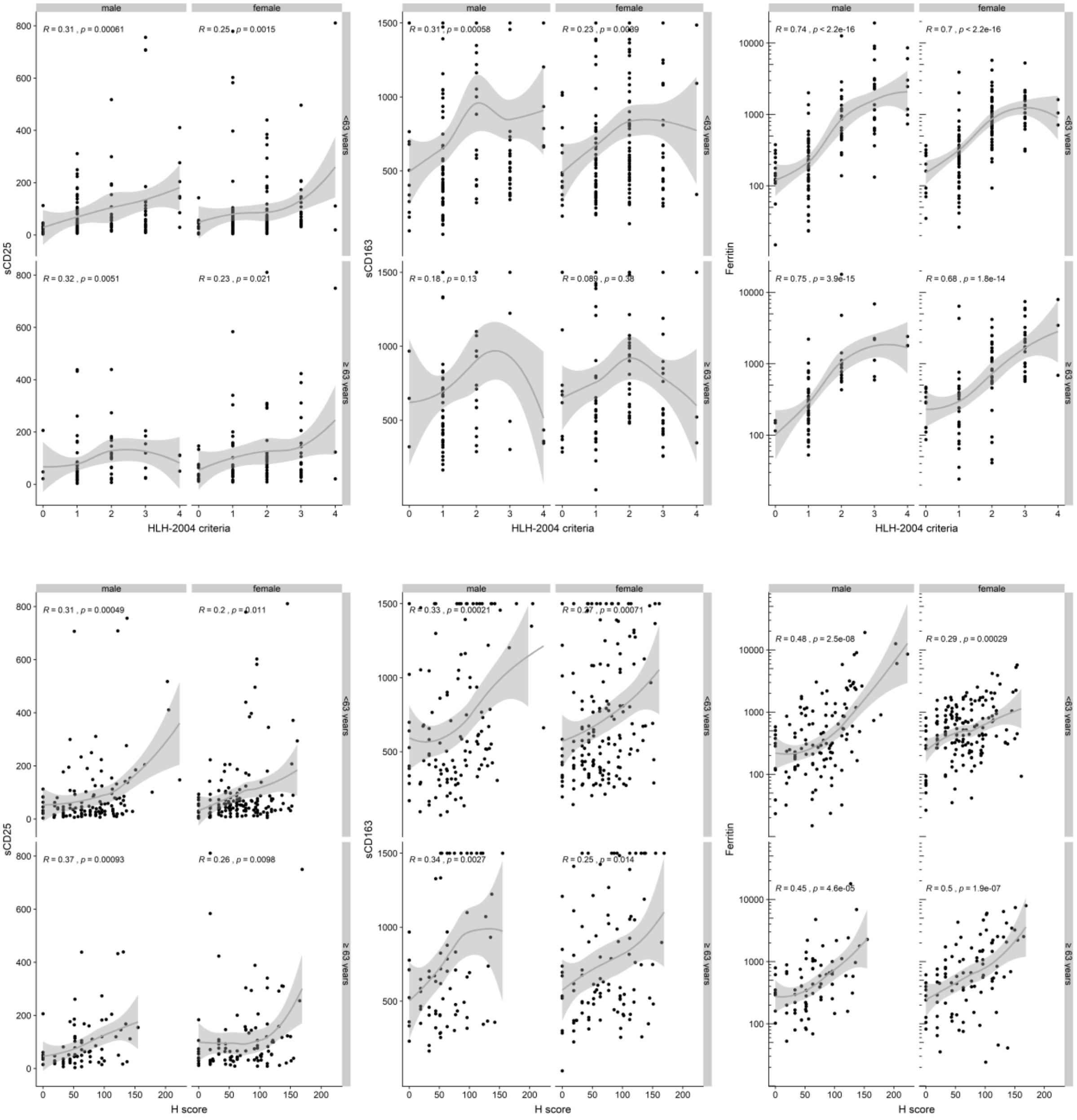

Figure S2. Age-and sex-dependent biomarker correlation to the studied scores.

Table S1. Overview of the HLH-2004 criteria [1] and the Hscore [2].

HLH-2004 criteria	H-Score
	**Known underlying immune suppression**
	0 (no) or 18 (yes)
	**Temperature**
	0 (<38.4), 33 (38.4–39.4), or 49 (>39.4)
**Fever**	**Organomegaly**
**Splenomegaly**	0 (no), 23 (hepatomegaly or splenomegaly), or 38
**Cytopenias (affecting ≥2 of 3 lineages in the peripheral blood):**	(hepato- and splenomegaly)
hemoglobin <90 g/L)	**Nr. of cytopenias**
Platelets <100 × 10^9/L	0 (1 lineage), 24 (2 lineages), or 34 (3 lineages)
Neutrophils <1.0 × 10^9/L	
**Hypertriglyceridemia and/or hypofibrinogenemia:**	**Hypertriglyceridemia (mmol/L)**
Fasting triglycerides ≥3.0 mmol/L (i.e., ≥265 mg/dL)	0 (<1.5), 44 (1.5–4), or 64 (>4)
Fibrinogen ≤1.5 g/L	**Hypofibrinogenemia (g/L)**
**Hemophagocytosis**	0 (>2.5) or 30 (≤ 2.5)
in bone marrow or spleen or lymph nodes	**Hemophagocytosis**
**Low or absent NK-cell activity**	in bone marrow aspirate
**Ferritin ≥500 mg/L**	0 (no) or 35 (yes)
**Soluble CD25 ≥2400 U/mL**	**Ferritin**
	0 (<2000), 35 (2000–6000), or 50 (>6000)
	**Serum glutamic oxaloacetic transaminase (IU/L)**
	0 (<30) or 19 (>30)

Table S2. Distribution of infections according to ECDC classification criteria modified ECDC class according to [3].

Type	ECDC Class	*n*	%
**Bloodstream infection[1]**	C-CVC[3] (*n* = 20), S-DIG[4] (*n* = 20), S-PUL[5] (*n* = 11), S-SSI[6] (*n* = 9), S-SST[7] (*n* = 3) S-UTI[8] (*n* = 12), S-OTH[9] (*n* = 18), S-UO[10] (*n* = 33)	126	39.7%
**Respiratory tract infection[2]**	PN1[11] (*n* = 3), PN3[12] (*n* = 6), PN4[13] (*n* = 5), PN5[14] (*n* = 62), LRI-Bron[15](*n* = 3), LRI-Lung[16](*n* = 2)	81	25.6%
**Gastrointestinal system infection[2]**	GI-CDI[17] (*n* = 4), GI-GE[18] (*n* = 4), GI-GIT[19] (*n* = 7), GI-IAB[20] (*n* = 13), EENT-ORAL[21] (*n* = 2)	30	9.5%
**Urinary tract infection[2]**	UTI-A[22] (*n* = 19), UTI-B[23] (*n* = 20)	39	12.3%
**Others[2]**	SYS-CESP[24] (*n* = 6), SYS-DI[25] (*n* = 7), SSI-S[26] (*n* = 9), SSI-D[27] (*n* = 1), CVS-Card[28] (*n* = 2), CVS-Vasc[29] (*n* = 1), CVS-Endo[30] (*n* = 2), SST-Skin[31] (*n* = 4), SST-ST[32] (*n* = 3), REPR-OREP[33] (*n* = 2), CRI-CVC[34] (*n* = 3), CNS-IC[35] (*n* = 1)	41	12.9%
**Total**		317	100%

Type = type of infection, [1]= blood culture positive; [2]= blood culture negative; [3]= blood stream infection (BSI), related to central vascular catheter; [4]= BSI, secondary digestive tract infection; [5]= BSI, secondary to pulmonary infection; [6]= BSI, secondary to surgical site infection; [7]= BSI, secondary to skin and soft tissue infection; [8]= BSI, secondary to urinary tract infection; [9]= BSI, secondary to another infection; [10]= BSI, (confirmed) unknown origin; [11]= pneumonia, positive quantitative culture from minimally contaminated lower respiratory tract specimen; [12]= pneumonia, microbiological diagnosis by alternative microbiology methods, [13]= pneumonia, positive sputum culture or non-quantitative culture from lower respiratory tract specimen; [14]= pneumonia, clinical signs of pneumonia without positive microbiology; bronchitis, tracheobronchitis, bronchiolitis, tracheitis, without evidence of pneumonia; [15]= LRI, other infections of the lower respiratory tract, bronchitis, tracheobronchitis, bronchiolitis, tracheitis;[16]= lower respiratory tract infection, other than pneumonia; [17]= gastrointestinal system infections (GI) *clostridium difficile* infection; [18]= GI, gastroenteritis (excluding CDI); [19]= Gastrointestinal tract (oesophagus, stomach, small and large bowel, and rectum), excluding GE, CDI; [20]= GI, intra-abdominal infection, not specified elsewhere; [21]= eye, ear, nose or mouth infection (EENT), oral cavity (mouth, tongue, or gums); [22]= urinary tract infection (UTI), microbiologically confirmed symptomatic UTI; [23]= UTI, not microbiologically confirmed symptomatic UTI; [24]= systemic infections (SYS), clinical sepsis in adults and children; [25]= SYS, disseminated infection; [26]= surgical site infection (SSI), superficial incisional; [27]= surgical site infection (SSI), deep incisional; [28]= cardiovascular system infection (CVS), myocarditis or pericarditis; [29] = CVS, arterial or venous infection; [30]= endocarditis, [31]= skin and soft tissue infections (SST), skin; [32]= SST, soft tissue (necrotizing fasciitis, infectious gangrene, necrotizing cellulitis, infectious myositis, lymphadenitis, or lymphangitis); [33]= reproductive tract infections (REPR)-other infections of the male or female reproductive tract (OREP), [34]= central vascular catheter-related infection (CRI), general CVC-related infection (no positive blood culture); [35]= central nervous system infection (CNS), intracranial infection.

Supplementary Methods

Bacteremia was defined by a positive BC result or real-time multiplex polymerase chain reaction (PCR) analysis result for a recognized bacterial species. Bacterial contaminants were specified as described in [3]. Coagulase-negative staphylococci (CNS) were recognized as causative pathogens only when found in two blood samples taken in separate occasions. Blood specimens were cultured in a set of FA Plus (aerobic) and FN Plus (anaerobic) bottles using the BacT/ALERT 3D automated blood culture system (bioMérieux, Marcy l'Etoile, France). Bacterial pathogens were identified by matrix-assisted laser desorption ionization (MALDI) time of flight (TOF) mass spectroscopy (MS) using

Microflex LT with the Biotyper database (Bruker Daltonik GmbH, Bremen, Germany). In the event of *Streptococcus pneumoniae* detection, the test result was further verified by optochin disc tests. In 220 patients, the occurrence of microbial DNA was additionally tested by the SeptiFast MGRADE assay, as described in [4]. Further, the occurrence of infection was assessed retrospectively by applying the definition criteria for hospital-acquired infections, established by the European Centre of Disease Control [3].

The following blood variables were analyzed on the first day after blood culture taking as standard laboratory parameters in an ISO 9001 certified and ISO 15189 accredited environment: procalcitonin (PCT, ng/ml, Hoffmann-La Roche Ltd, Basel, Switzerland), C-reactive protein (CRP, mg/dl, Latex test; Beckman Coulter, Brea, CA, USA), fibrinogen according to Clauss (Fib, mg/dl, Hoffmann-La Roche Ltd, Basel, Switzerland), aspartate transaminase (ASAT, U/L, Beckman Coulter), triglycerides (mg/dl, Beckman Coulter), and ferritin (ng/ml, Beckman Coulter). Variables of the complete blood count including white blood cell counts (WBC, G/l), hemoglobin (Hb, g/dl), platelets (G/l), and relative proportion of neutrophils (NeuR, %) were analyzed using a Stromatolyser-4DS (Sysmex, Norderstedt, Germany).

References

1. Henter, J.-I.; Horne, A.; Aricó, M.; Egeler, R.M.; Filipovich, A.H.; Imashuku, S.; Ladisch, S.; McClain, K.; Webb, D.; Winiarski, J.; et al. HLH-2004: Diagnostic and therapeutic guidelines for hemophagocytic lymphohistiocytosis. *Pediatr. Blood Cancer* **2007**, *48*, 124–131.
2. Fardet, L.; Galicier, L.; Lambotte, O.; Marzac, C.; Aumont, C.; Chahwan, D.; Coppo, P.; Hejblum, G. Development and validation of the HScore, a score for the diagnosis of reactive hemophagocytic syndrome. *Arthritis Rheumatol Hoboken NJ* **2014**, *66*, 2613–2620.
3. European Centre for Disease Prevention and Control. Point prevalence survey of healthcare-associated infections and antimicrobial use in European acute care hospitals protocol version 4.3, full-scale survey and codebook. Stockholm: ECDC; 2012.
4. Ratzinger, F.; Tsirkinidou, I.; Haslacher, H.; Perkmann, T.; Schmetterer, K.G.; Mitteregger, D.; Makristathis, A.; Burgmann, H. Evaluation of the Septifast MGrade Test on Standard Care Wards-A Cohort Study. *PloS One* **2016**, *11*, e0151108.
